# Supplementary material for: Genome-Wide Association Study Reveals the Genetic Basis of Kernel and Cob Moisture Changes in Maize at Physiological Maturity Stage
Source: Plants (Basel). 2022 Jul 30;11(15):1989. doi: 10.3390/plants11151989 (PMC9370647; doi:10.3390/plants11151989)
Supplement: Supplementary file 1 [file plants-11-01989-s001.zip › plants-1832550-supplementary.pdf]

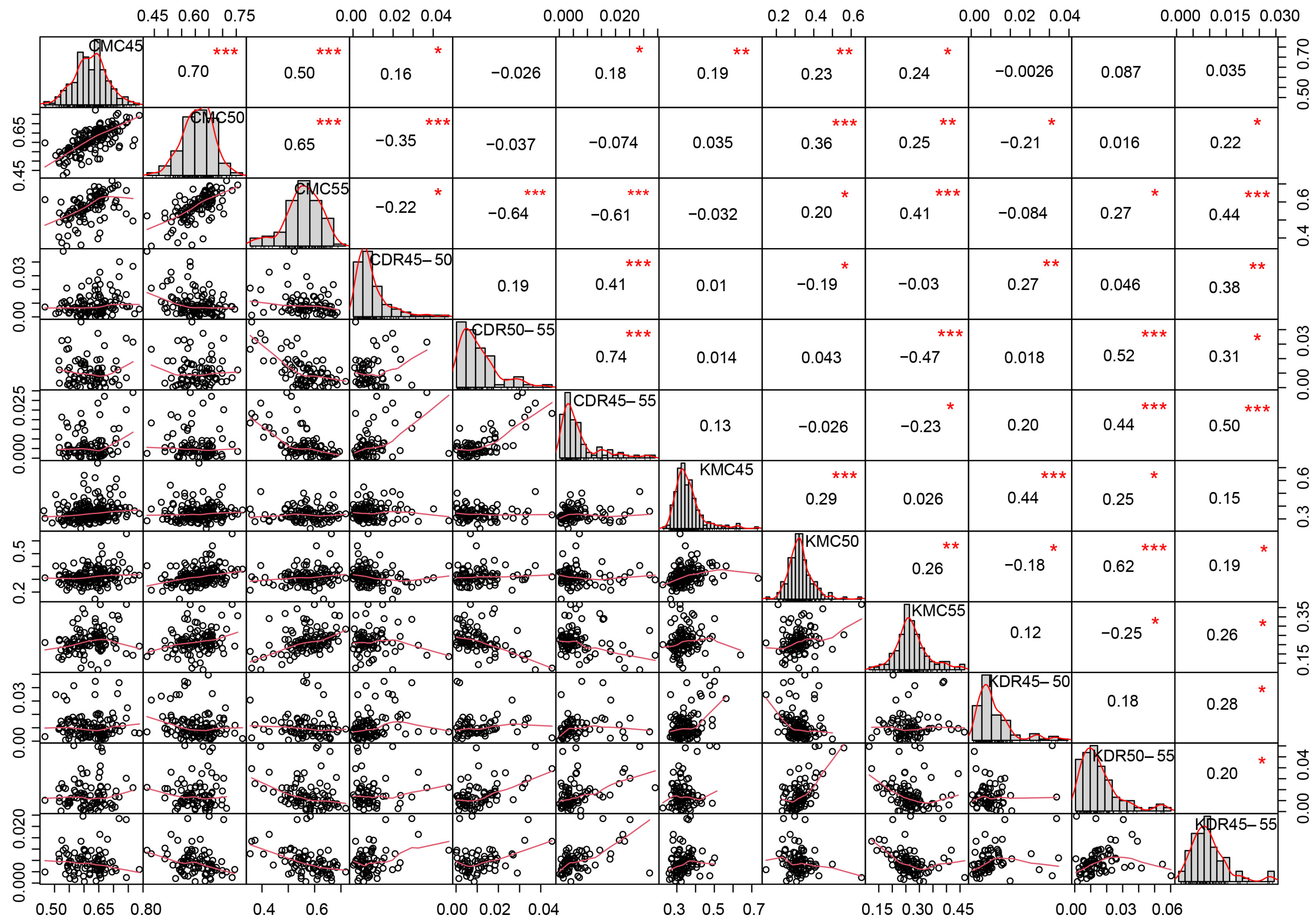

Figure S1 The phenotypic distributions of 12 collected traits at different time points. The CMC45, CMC50, and CMC55 represent cob moisture content on the 45th, 50th, and 55th days after pollination, respectively. The CDR45–50, CDR50–55, and CDR45–55 represent cob dehydration rate of three time spans: 45–50, 50–55, and 45–55 days after pollination, respectively. The KMC45, KMC50, and KMC55 represent kernel moisture content on the 45th, 50th, and 55th days after pollination, respectively. The KDR45-50, KDR50–55, and KDR45–55 represent kernel dehydration rate of three time spans: 45–50, 50–55, and 45–55 days after pollination, respectively. *P = 0.05, **P = 0.01, ***P = 0.001.

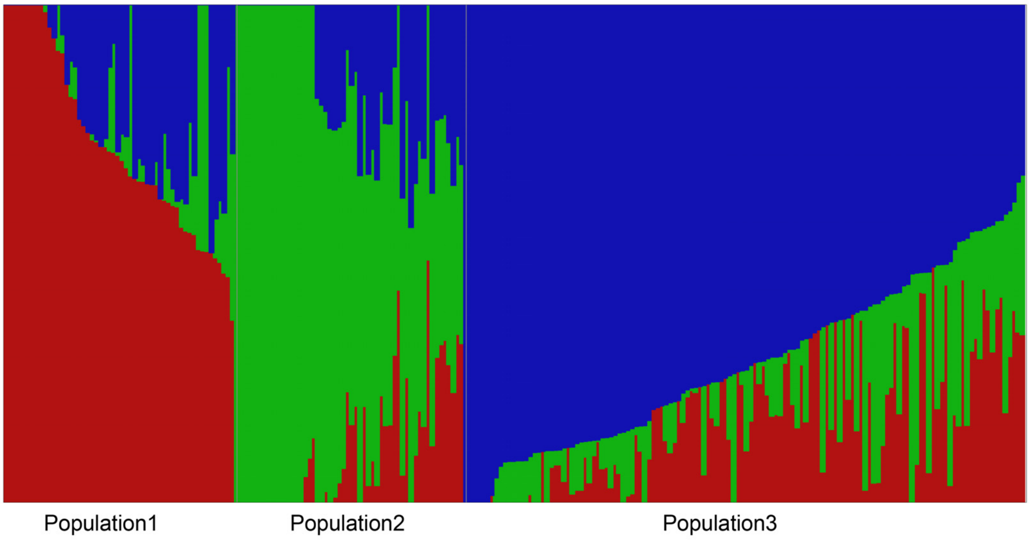

Figure S2 Population structure of 241 maize inbred lines estimated from 46,603 SNPs. Population 1, population 2, and population 3 represent tropical group, non-stiff stalk (NSS) group, and stiff stalk (SS) group, respectively.

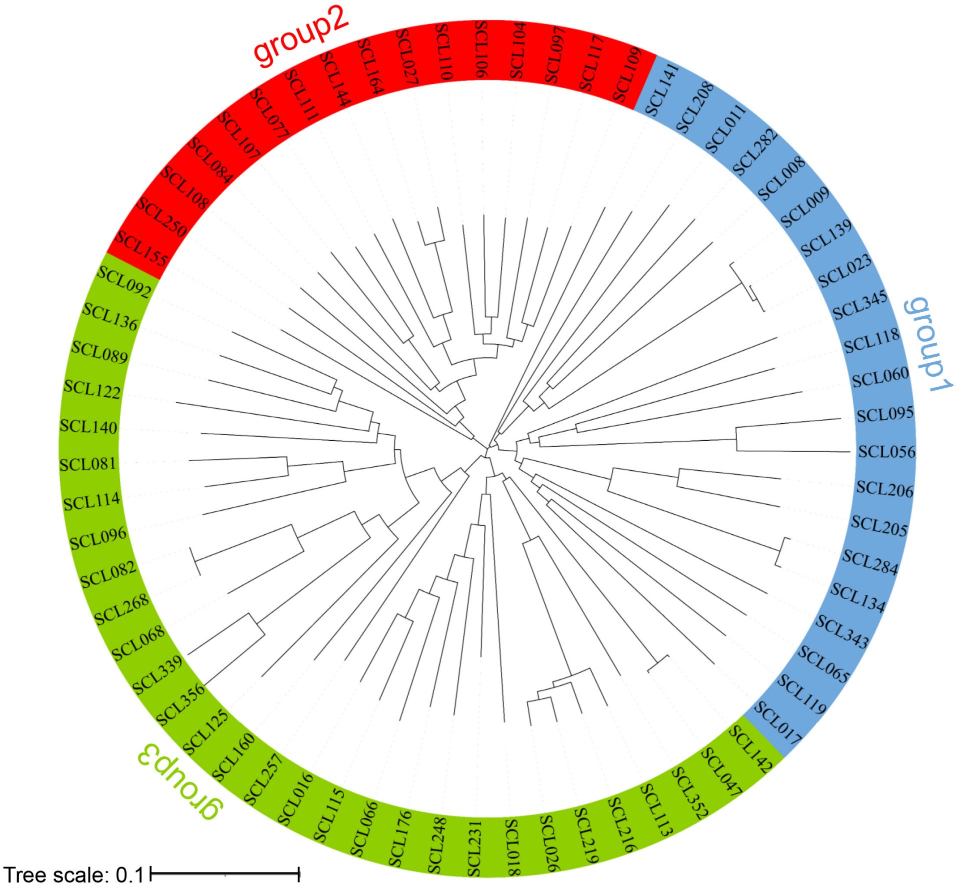

Figure S3 Phylogenetic tree of 67 maize inbred lines. The group 1, group 2, and group 3 represent tropical group, non-stiff stalk (NSS) group and stiff stalk (SS) group, respectively.

Table S1 Significant SNPs detected by GWAS using FarmCPU

Trait	SNP	Chromosome	Position (bp)	P-value	Effect
CDR45–55	SYN38588	7	121316500	9.58E-11	-0.005
	SYN11290	2	70284631	1.8E-09	-0.005
	SYN30412	5	20908120	1.37E-07	-0.003
	PZE-103097076	3	156937084	6.1E-07	0.003
CDR50–55	SYN38588	7	121316500	2.1E-09	-0.005
	PZE-102053209	2	30811954	2.39E-09	0.005
	PZE-101122473	1	152107078	4.9E-09	0.004
KMC45	PZB01400.1	1	286449514	3.19E-07	0.031
	SYN15586	1	280706296	5.87E-07	0.041
	SYN8680	1	283601391	9.87E-07	0.033

CDR45–55 and CDR50–55 represent cob dehydration rate on the 45–55 and 50–55 days after pollination, respectively. KMC45 represent kernel moisture content on the 45th days after pollination.

Table S2 Annotations of candidate genes based on B73 (RefGen_v4) genome.

Trait	Candidate Gene	Chromosome	Annotation
CDR45–55	*Zm00001d003958*	2	-
	Zm00001d003957	2	-
	Zm00001d003950	2	major facilitator superfamily protein
	Zm00001d003962	2	-
	Zm00001d003961	2	-
	Zm00001d013818	5	tyrosine N-monooxygenase
	Zm00001d013820	5	-
	Zm00001d013814	5	pentatricopeptide repeat-containing protein
	Zm00001d038319	5	COV1-like protein
	Zm00001d013816	5	DTW domain-containing protein
	Zm00001d013828	5	-
	Zm00001d013824	5	-
	Zm00001d013819	5	-
	Zm00001d013817	5	-
	Zm00001d013825	5	-
	Zm00001d038318	5	-
	Zm00001d013812	5	polyadenylate-binding protein RBP45C
	Zm00001d042276	3	-
	Zm00001d042281	3	-
	Zm00001d042279	3	-
	Zm00001d042269	3	DNAJ heat shock N-terminal domain-containing protein
	Zm00001d042274	3	-
	Zm00001d042268	3	receptor-like protein kinase 5
	Zm00001d042275	3	phosphatidylinositol transfer protein CSR1
	Zm00001d042271	3	-
	Zm00001d042272	3	histone-lysine N-methyltransferase family protein
	Zm00001d042270	3	heavy metal transport/detoxification superfamily protein
	Zm00001d042284	3	-
	Zm00001d042278	3	phytanoyl-CoA dioxygenase 2
CDR50–55	*Zm00001d003107*	2	-
	Zm00001d003109	2	alpha-12-glucosyltransferase
	Zm00001d003108	2	adenine phosphoribosyltransferase 1

	Zm00001d003104	2	srotein binding protein
	Zm00001d003099	2	serinc-domain containing serine and sphingolipid biosynthesis protein
	Zm00001d003102	2	cytokinin-O-glucosyltransferase 3
	Zm00001d003103	2	-
	Zm00001d003101	2	-
	Zm00001d003106	2	-
	Zm00001d003110	2	-
	Zm00001d030697	1	-
	Zm00001d030698	1	-
KMC45	*Zm00001d034383*	1	-
	Zm00001d034388	1	indole-3-acetaldehyde oxidase-like
	Zm00001d034386	1	GATA transcription factor 2-like
	Zm00001d034396	1	mavicyanin
	Zm00001d034400	1	-
	Zm00001d034399	1	-
	Zm00001d034379	1	-
	Zm00001d034380	1	-
	Zm00001d034387	1	-
	Zm00001d034401	1	chloroplastic zinc metalloprotease EGY1
	Zm00001d034382	1	-
	Zm00001d034188	1	cold-regulated 413 plasma membrane protein 2
	Zm00001d034175	1	-
	Zm00001d034189	1	CASP-like protein 12
	Zm00001d034180	1	seven transmembrane domain protein
	Zm00001d034186	1	allene-oxide synthase 3
	Zm00001d034181	1	-
	Zm00001d034167	1	-
	Zm00001d034179	1	nuclear pore complex protein NUP58
	Zm00001d034164	1	plastoquinol-plastocyanin reductase
	Zm00001d034183	1	serine/threonine-protein kinase 12
	Zm00001d034178	1	-
	Zm00001d034184	1	pyridoxal phosphate (PLP)-dependent transferase superfamily
	Zm00001d034182	1	allene-oxide synthase 4
	Zm00001d034173	1	-
	Zm00001d034301	1	-
	Zm00001d034279	1	-

Zm00001d034305	1	-
Zm00001d034298	1	transcription factor phytochrome interacting factor-like 13
Zm00001d034283	1	photosystem I reaction center subunit III
Zm00001d034312	1	-
Zm00001d034286	1	-
Zm00001d034314	1	transcription initiation factor TFIID subunit 7
Zm00001d034313	1	seed maturation protein
Zm00001d034307	1	-

CDR45–55 and CDR50–55 represent cob dehydration rate on the 45–55 and 50–55 days after pollination, respectively. KMC45 represents kernel moisture content on the 45th, 50th, and 55th days after pollination, respectively. '-' represents no functional annotations.

Table S3 Homologous annotations of 11 unknown genes

Candidate gene	Homologous gene	Annotation
Zm00001d020618	*OSNPB_090439700*	transmembrane protein 87A (*Oryza sativa*)
Zm00001d020610	*OSNPB_090438000*	respiratory burst oxidase homolog protein E (*Oryza sativa*)
Zm00001d020609	-	-
Zm00001d020612	*OSNPB_090438400*	probable ethanolamine kinase (*Oryza sativa*)
Zm00001d020622	-	-
Zm00001d020626	-	-
Zm00001d020613	*PVAP13_2NG338900*	translation initiation factor IF-2-like (*Panicum virgatum*)
Zm00001d020614	*SORBI_3002G214800*	bicolor protein TIFY 10c (*Sorghum bicolor*)
Zm00001d020616	-	-
Zm00001d020627	*OSNPB_090441000*	subtilisin-like protease precursor (*Oryza sativa*)
Zm00001d020620	-	-

'-' represents no homologous or annotations.

Table S4 Details of 38 variations detected in two genes

Gene	Marker	Variation Position	Variation Type
Zm00001d020615	S7_125162271	125162271	G/A
	S7_125162276	125162276	G/A
	S7_125162319	125162319	C/A
	S7_125162440	125162440	T/C
	S7_125162448	125162448	A/T
	S7_125162603	125162603	G/A
	S7_125162646	125162646	C/T
	S7_125162869	125162869	-/C
	S7_125162886	125162886	C/T
	S7_125163651	125163651	C/-
	S7_125164549	125164549	T/C
	S7_125164562	125164562	A/G
	S7_125164643	125164643	C/T
	S7_125165041	125165041	T/C
	S7_125165056	125165056	T/C
	S7_125165191	125165191	G/A
	S7_125165529	125165529	T/C
	S7_125165553	125165553	T/C
	S7_125165963	125165963	A/G
	S7_125166026	125166026	G/C
	S7_125166053	125166053	C/T
	S7_125166137	125166137	G/A
	S7_125166170	125166170	G/T
	S7_125166242	125166242	C/T
	S7_125166344	125166344	A/G
	S7_125166395	125166395	A/G
	S7_125166437	125166437	G/A
	S7_125166495	125166495	C/A
	S7_125166940	125166940	C/T
Zm00001d020623	S7_125319616	125319616	A/C
	S7_125320422	125320422	G/A
	S7_125320782	125320782	C/T
	S7_125321596	125321596	C/T
	S7_125321635	125321635	A/G
	S7_125321649	125321649	C/T
	S7_125321749	125321749	C/G
	S7_125321983	125321983	T/G
	S7_125322584	125322584	T/-

'-' represents deletion.

Table S5 Significant SNPs overlapping with QTL for kernel moisture change identified in previous studies

SNP	Trait	Associated QTL (Marker)	Distance between SNP and QTL (Kb)	QTL Function	Reference
SYN38588	CDR45–55	PZE-107014666/PZE-107082484	–	m_{ini}	Yin et al. [11]
SYN30412	CDR45–55	SYN30432/PZE-105040767	80.00	m_{fni}	Yin et al. [11]
SYN38588	CDR50–55	PZE-107014666/PZE-107082484	–	m_{ini}	Yin et al. [11]
PZE-101122473	CDR50–55	PZE-101120411/PZE-101128157	–	t_{ini}	Yin et al. [11]
PZB01400.1	KMC45	PZE-101235343/SYN25670	–	m_{ini}	Yin et al. [11]
		SNP_286449826	0.31	KMC 40 DAP	Li et al. [25]
SYN8680	KMC45	PZE-101235343/SYN25670	–	m_{ini}	Yin et al. [11]

CDR45–55 and CDR50–55 represent cob dehydration rate on the 45–55 and 50–55 days after pollination, respectively. KMC45 represents kernel moisture content on the 45th days after pollination. m_{ini}, dehydration initial moisture. m_{fni}, final moisture. t_{ini}, dehydration initial time (DAP). KMC 40 DAP, kernel moisture content on the 40th days after pollination. "–" represents SNP located within the QTL.

Table S6 Information of 241 maize inbred lines in the association panel

Taxa	Name	Pedigree
SCL001	F19	Derived from CIMMYT germplasm
SCL003	Zheng58	Ye478 mutant
SCL004	Chang7-2	Wei95 × Huangzaosi // S901
SCL005	S273	Derived from suwan germplasm
SCL006	Su1611	Derived from suwan germplasm
SCL008	5311	Derived from suwan germplasm
SCL009	CTL26	Derived from suwan germplasm
SCL011	21A	Derived from Tropical germplasm
SCL014	V37	V37
SCL015	Mian7317	Mian7317
SCL016	Mian723	P953 × 698-3
SCL017	7041-5	Derived from Yogoslavic hybrid SC704
SCL018	NZ013-1	Neizi013-1-1
SCL019	Yu561	Nongda202 × 095
SCL020	H08-155	H08-155
SCL021	K169R	Derived from American hybrid 3163
SCL023	M14	M14
SCL025	B151	Derived from Pioneer unknown hybrid
SCL026	618	Derived from Pioneer unknown hybrid
SCL027	141	Derived from Pioneer hybrid 78599
SCL028	ShuangM9	M9 × M9B
SCL029	P953	Derived from hybrid 95-3
SCL030	LN8	478 improved line
SCL031	HZ127-7	Derived from American hybrid Y8G61
SCL033	ZYDH381-1	ZYDH381-1
SCL035	De2010-1	De2010-1
SCL036	End28	78599 improved lines
SCL038	Mian715	Hybrid CGT-15
SCL039	BS08-565	BS08-565
SCL042	De12	De12
SCL043	TD1	478 improved line
SCL045	Jing07-4	Qujing landrace
SCL046	M89	K22 improved line
SCL047	H127RE	American hybrid Y8G61
SCL049	NZ925	NZ925
SCL050	BS1074	BS1074
SCL051	Lin-1	478 improved line
SCL054	S7913	S7913
SCL055	DH29	Derived from Pioneer hybrid 78599
SCL056	Mo17	C103 × 187-2
SCL059	2142	Foreign hybrid Y7864
SCL060	205-11	Foreign hybrid Y7865F2 × Qi205

SCL065	Nan09530	095 × Zi330
SCL066	98-3	698-3 × 81565 // 698-3
SCL067	M232	65232/P6C3 // 65232
SCL068	Du321	American hybrid 3382
SCL070	Nan381	Ai351/B9001 // Ai351BC2
SCL071	7854	Ye478 × 156
SCL073	ZY2247	ZY2247
SCL074	Q78	Q78
SCL075	Nan637	Nan637
SCL076	Nan202	Zong3 / Niu2-1
SCL077	78599-211	Derived from Pioneer hybrid 78599
SCL078	YA3237	Zheng32 × S37
SCL079	JS0251	Dan85566/E28 second circle line
SCL081	Zheng28	Zheng28
SCL082	Zheng22	E28/Duqing second circle line
SCL084	Dan3130	Derived from Pioneer hybrid 78599
SCL085	LC955	LC955
SCL086	Dan599	Derived from Pioneer hybrid 78599
SCL087	LJ-2	American hybrid Liaojian
SCL088	Liao68	Unknown
SCL089	Liao7996	7922 / D9046
SCL090	Liao3053	(7922 × B68) / 5003
SCL092	Liao7890	7922 / 8001
SCL094	Wa138	Luda Red bone germplasm
SCL095	Ji1037	Mo17 / suwan1 // MO17
SCL096	TS6278	TS6278
SCL097	ZM28	ZM28
SCL099	K22	K11 × Ye478
SCL100	Dan9046	7922 × 5003
SCL101	PHB09	American inbred line， Reid population
SCL102	CN9802	American inbred line， Reid population
SCL103	Dan598	Dan598
SCL104	Shen135	Derived from Pioneer hybrid 78599
SCL105	Shen136	Derived from Pioneer hybrid 78599
SCL106	Shen137	Derived from Pioneer hybrid 6JK111
SCL107	SH15	Derived from Pioneer hybrid 78599
SCL108	871	Derived from Pioneer hybrid 87001
SCL109	P138	Derived from Pioneer hybrid 78599
SCL110	Qi319	Derived from Pioneer hybrid 78599
SCL111	178	Derived from Pioneer hybrid 78599
SCL112	7327	7327
SCL113	YS0	YS0
SCL114	Ye478	U8112 × Shen5003
SCL115	698-3	Derived from Pioneer hybrid 78599

SCL116	JH961	Derived from Pioneer hybrid 78599
SCL117	JH59	Derived from Pioneer hybrid 78599
SCL118	Qi205	vAi141 × Zhongxi017 / QPM70
SCL119	Dan340	White bone Lu9 × glume maize
SCL120	CA211	CA211
SCL121	DYS	DYS
SCL122	Liao6082	5003、7922、U8112、Zheng32、N-46、B73 integrative line
SCL123	Lu2458	Lu2458
SCL124	Ji477	Mo17 radiation
SCL125	Zheng29	Zheng29
SCL126	ZH64	Derived from Pioneer hybrid 64
SCL127	H921	78599 × (6166 + 6066 + 6165)
SCL128	1572	5003 × Luda Red bone line
SCL129	Ji992	Ji992
SCL130	9614	Tropical germplasm
SCL131	434	434
SCL132	H10	Tropical germplasm
SCL133	4379	4379
SCL134	4011	Derived from landrace
SCL135	HuangC	(Huangxiao162 × Zi330 / 02) × Tuxpeno
SCL136	Tie7922	American hybrid 3382
SCL139	H21	Huangzaosi × H84
SCL140	4866	Ye478 improved line
SCL141	07G83	07G83
SCL142	LJS-1	LJS-1
SCL144	LZM004	Tropical germplasm
SCL147	LZM025	Tropical germplasm
SCL148	W8071	Tropical germplasm
SCL151	QBII-1	1221CL13
SCL153	ML1108	ML1108
SCL154	ML1120	ML1120
SCL155	W7475	12127475
SCL157	PN0504-8	Derived from Pioneer hybrid PN0504
SCL158	KS001	Derived from American hybrid X1141P
SCL160	5220-2	Unknown
SCL162	SAM3001	Unknown hybrid 3001
SCL163	975-12	698-3 / unknown inbred line
SCL164	18-599	Derived from Pioneer hybrid 78599
SCL166	SCML1950	American hybrid 441950
SCL167	08-641	Derived from Pioneer hybrid Y78641
SCL168	48-2	(Mo17 / Dan340)// 6 unknown lines
SCL169	SCML103	698-3 / unknown line
SCL171	SCML203	(48-2 × 5003)space induced mutation × foreign hybrid
SCL172	LH8012	Yunrui9 (YML46 / YML8711) second circle line

SCL174	TM012	TM012
SCL175	1323	Hybrid 1323
SCL176	975-13	698-3 / unknown line
SCL178	SCML2054	Hybrid 2054
SCL179	10WRB115	10WRB115
SCL180	10WRA120	10WRA120
SCL181	08WSC51	08WSC51-221
SCL182	08WSC179	08WSC179-31
SCL183	08WSC187	08WSC187-211
SCL184	08WSC200	08WSC200-11
SCL185	08WSC204	08WSC204-21
SCL186	08WSC237	08WSC237-111
SCL187	08WSC257	08WSC257-122
SCL189	CIMMYT-1	CIMMYT-1
SCL191	GP66-1	Unknown
SCL192	S37	Derived from suwan1
SCL195	BJ005	BJ005
SCL196	2082-11	2082-11
SCL197	GD003	11GD003
SCL199	XS021	XS021
SCL200	9HT1736	Unknown
SCL204	LH7556	LH7556
SCL205	Mian04185-4	Mian04185-43122
SCL206	Mian04185-8	Mian04185-8411
SCL208	SCML2031	TF2031-2221
SCL209	TY30331-3	TY30331-35121
SCL210	TY30331-2	TY30331-25111
SCL211	QA	178/9782
SCL212	KS003	Derived from integrative line
SCL213	HL5049	HL5049
SCL214	HL5054	HL5054
SCL215	LLF-08	08-641 backcross improved line
SCL216	ZD808-1	Hybrid ZD808
SCL217	510317	Hybrid 510317
SCL218	Dan4245	Liao7980 / Dan598
SCL219	PZ1010-2	PZ1010-2
SCL221	JD7275	Hybrid JD7275
SCL222	JY01-3	Hybrid JY01
SCL223	Y0827	SN203 / SN204 / SC9911 / DK656 / CS9906 integrative line
SCL227	Y1021	Ye478 / 5003 / 7922 / Du32 / Cheng687 / 698-3 integrative line
SCL228	Y1022	Ye478 / 5003 / 7922 / Du32 / Cheng687 / 698-3 integrative line
SCL229	Y1032W	273 / CH01-1112 / 634-1112 / 698-3 / Cheng687 / 7932-1211 / 5003 / 18-599 / Hai92-1 integrative line

SCL230	Y1032R	273 / CH01-1112 / 634-1112 / 698-3 / Cheng687/7932-1211 / 5003 / 18-599 / Hai92-1 integrative line
SCL231	Y1035	08-641 natural abnormal plant
SCL232	Y1038	08-641 natural abnormal plant
SCL233	Y1111	Y1111
SCL235	Y1027	Derived from integrative line W1030
SCL240	77	77
SCL243	10WRC64	10WRC64
SCL245	PI43W	PI43W
SCL246	W8199	11278199
SCL247	Y1114	Tropical integrative line AP5
SCL248	LX312	LX312
SCL250	DH3732	Denghai hybrid DH3732
SCL253	BML1269	BML1269
SCL254	Y1216	Y1216
SCL255	LM-6	LM-6
SCL257	BML1243	BML1243
SCL258	BML1234	BML1234
SCL259	Y1224	Y1224
SCL260	1217 8107	1217 8107
SCL261	BML1228	BML1228
SCL262	08WSC166	08WSC166-1211
SCL263	LX350	LX350
SCL264	Y1217	Y1217
SCL265	XBY2193	SUWAN germplasm
SCL268	U8112	Hybrid3382
SCL272	LS-22	LS-22
SCL273	CD30M	Hybrid CGT-15
SCL275	XH05	Yunnan White XH05 × American hybrid
SCL276	06WAM210	CIMMYT line
SCL281	GCML152	CIMMYT line
SCL282	GCML157	CIMMYT line
SCL283	MX714	YA3237 / CIMMYT hybrid
SCL284	DH40	DH40
SCL289	CLWN201	CIMMYT line
SCL304	CML282	CIMMYT line
SCL307	CML379	CIMMYT line
SCL310	TL98A1709-20	CIMMYT line
SCL311	TL96B	CIMMYT line
SCL318	BANTAN2003	CIMMYT line
SCL319	CG698C102	698-3 improved line
SCL320	CG921	Hai921 improved line
SCL322	Lian87	Lian87

SCL324	K305	American hybrid 2021
SCL325	811	Derived from 136-87mutant
SCL326	K363	48-2 × 156
SCL330	81565	Miandan 8 parent Mobai
SCL332	WZ-1	W1 Wanzhou
SCL333	PB80	1067-1 × B73 / B73Ht.1 BC6, SS
SCL335	793	235 / B73, SS
SCL337	PHV63	555 × Zap < 4CB, NSS
SCL338	PHW65	(861 / 595) × 8211131X,NSS
SCL339	2369	2702H × B73) × B73, SS
SCL341	PHW52	(B73 / G39) × 411242X, SS
SCL342	CA1108	Huangzaosi improved line
SCL343	Nan21-3	Yogoslavic hybrid BC8241Ht
SCL344	PH6WC	PH01N × PH09B, Reid population
SCL345	PH4CV	PH7V0 × PHBE2，Lancaster population
SCL346	Xun9058	6JK × 8085 Thailand
SCL347	LX9801	502 × H21
SCL348	C24	C24
SCL349	C09-1	09-1
SCL351	ZNC-4	Unknown
SCL352	Qi533	Derived from P136 abnormal plant
SCL353	L6201	08-641 improved line
SCL354	LSC127	LSC127
SCL355	W30	K22 improved line
SCL356	B73	BSSS C5
SCL358	W3189	18-599 improved line
SCL359	9782	(48-2 × 5003) space induced mutant × foreign hybrid line
